# Strengthening the incentives for responsible research practices in Australian health and medical research funding

**DOI:** 10.1186/s41073-021-00113-7

**Published:** 2021-08-02

**Authors:** Joanna Diong, Cynthia M. Kroeger, Katherine J. Reynolds, Adrian Barnett, Lisa A. Bero

**Affiliations:** 1 School of Medical Sciences, Faculty of Medicine and Health, The University of Sydney, Sydney, NSW 2006 Australia; 2 Charles Perkins Centre, The University of Sydney, Sydney, NSW 2006 Australia; 3 Central Clinical School, Faculty of Medicine and Health, The University of Sydney, Sydney, NSW 2006 Australia; 4 School of Public Health, Faculty of Medicine and Health, The University of Sydney, Sydney, NSW 2006 Australia; 5 Australian Centre for Health Services Innovation and Centre for Healthcare Transformation, School of Public Health and Social Work, Faculty of Health, Queensland University of Technology, QLD, Brisbane, 4000 Australia; 6 School of Pharmacy, Faculty of Medicine and Health, The University of Sydney, Sydney, NSW 2006 Australia; 7 Centre for Bioethics and Humanities, University of Colorado, Boulder, CO 80309-0552 USA

**Keywords:** Grant, Fellowship, Medical, Research transparency, Research reproducibility, Research integrity

## Abstract

**Background:**

Australian health and medical research funders support substantial research efforts, and incentives within grant funding schemes influence researcher behaviour. We aimed to determine to what extent Australian health and medical funders incentivise responsible research practices.

**Methods:**

We conducted an audit of instructions from research grant and fellowship schemes. Eight national research grants and fellowships were purposively sampled to select schemes that awarded the largest amount of funds. The funding scheme instructions were assessed against 9 criteria to determine to what extent they incentivised these responsible research and reporting practices: (1) publicly register study protocols before starting data collection, (2) register analysis protocols before starting data analysis, (3) make study data openly available, (4) make analysis code openly available, (5) make research materials openly available, (6) discourage use of publication metrics, (7) conduct quality research (e.g. adhere to reporting guidelines), (8) collaborate with a statistician, and (9) adhere to other responsible research practices. Each criterion was answered using one of the following responses: “Instructed”, “Encouraged”, or “No mention”.

**Results:**

Across the 8 schemes from 5 funders, applicants were instructed or encouraged to address a median of 4 (range 0 to 5) of the 9 criteria. Three criteria received no mention in any scheme (register analysis protocols, make analysis code open, collaborate with a statistician). Importantly, most incentives did not seem strong as applicants were only instructed to register study protocols, discourage use of publication metrics and conduct quality research. Other criteria were encouraged but were not required.

**Conclusions:**

Funders could strengthen the incentives for responsible research practices by requiring grant and fellowship applicants to implement these practices in their proposals. Administering institutions could be required to implement these practices to be eligible for funding. Strongly rewarding researchers for implementing robust research practices could lead to sustained improvements in the quality of health and medical research.

**Supplementary Information:**

The online version contains supplementary material available at 10.1186/s41073-021-00113-7.

## Background

Australian health and medical research funders support substantial research efforts in health and biomedical research. Each year, $7.9 billion (4% of all spending on health) is spent on health and medical research in Australia, with funds channelled through the National Health and Medical Research Council (NHMRC, $845 million), the Australian Research Council’s contribution to health and medical research (ARC, $79 million), and the Medical Research Future Fund (MRFF, $392 million) [1]. Research grants and fellowships are usually awarded by merit on a competitive basis. The ability to secure funding can potentially make or break research careers by developing research programs and teams or impeding their development [2, 3]. Consequently, incentives in grant funding schemes substantially influence the behaviour of researchers [4].

Health and medical research funders, and the scientific community as a whole, recognise that the quality of published research and current reporting practices can be inadequate. Research findings can be poorly reported or at high risk of bias, as highlighted in the ongoing COVID-19 pandemic [5, 6], so ensuring research rigour is especially important. Funders such as the NHMRC and the ARC have called for stronger emphasis on responsible research practices, methodological rigor, and transparency [7, 8]. However, it is not known if these calls translate to incentives for responsible research practices in funding applications. This is important, because funders significantly influence research culture and could positively impact researchers’ behaviour. Thus, we conducted an audit of instructions from research grant and fellowship schemes to determine to what extent Australian health and medical funders incentivise responsible research practices.

## Method

Category 1 to 3 [9] research grants and fellowships were purposively sampled from the 2018 Australian Competitive Grants Register [10]. Category 1 grants are Australian competitive grant research and development (R&D) income. Category 2 grants are other public sector R&D income, including grants from local, state or partial government bodies. Category 3 grants are industry and other R&D income, including grants from the private sector, philanthropic and international sources.

Funding schemes were sampled to select government and non-profit schemes that awarded the largest amount of funds, as identified by The University of Sydney (Research Grants and Contracts: https://www.sydney.edu.au/research/research-funding.html) at the time of the audit (Sep 2019). These schemes were selected because they are highly competitive and might influence the behaviour of many researchers. For funders with multiple schemes, recurring investigator-initiated schemes were sampled. Schemes were excluded if they were special calls, internal schemes, international funding, or partnership grants. The following 8 schemes were assessed: ARC Discovery Projects, ARC Discovery Early Career Researcher Award (DECRA), NHMRC Project Grants, NHMRC Career Development Fellowships (CDF), NHMRC Clinical Trials and Cohort Studies (CTCS) Grants, Diabetes Australia Research Trust (DART) General Grants and Millennium Awards, National Breast Cancer Foundation (NBCF) Investigator Initiated Research Scheme, Bupa Foundation (Australia) Limited Bupa Health Foundation grants.

Nine criteria were developed to assess instructions from the schemes. We focused on incentives for responsible research practices based on principles from large consensus discussions on assessing scientists [11] and the Transparency and Openness Promotion Guidelines [12]. These principles are important as they are in keeping with principles of research integrity, research rigour, and the Australian Code for the Responsible Conduct of Research [13]. Our criteria determined if instructions from the schemes incentivise applicants to: (1) publicly register study protocols before starting data collection, (2) register analysis protocols before starting data analysis, (3) make study data openly available, (4) make analysis code openly available, (5) make research materials openly available, (6) discourage use of publication metrics, (7) conduct quality research (e.g. adhere to reporting guidelines), (8) collaborate with a statistician, and (9) adhere to other responsible research practices. If a scheme stipulated applicants to comply with supporting governance documents (e.g. Australian Code for the Responsible Conduct of Research 2007 [13]), data were extracted from the supporting documents.

Each criterion was answered using one of the following responses: “Instructed”, “Encouraged”, or “No mention”. Data were extracted by one investigator (KR) using a customised form in REDCap electronic data capture tools, and independently checked by another investigator (JD, CK). Differences were resolved by discussion. The questions to assess the 9 criteria and their interpretation are as follows:

1. *Do instructions incentivise publicly registering study protocols before starting data collection?*
  Instructions must state that study protocols must be publicly registered with a date and time-stamp before starting data collection.
2. *Do instructions incentivise registering analysis protocols before starting data analysis?*
  Instructions must state that analysis protocols must be registered with a date and time-stamp before starting data collection.
3. *Do instructions incentivise making study data openly available to the research community?*
  Instructions must state that data must be made openly or publicly available.
4. *Do instructions incentivise making analysis code openly available to the research community?*
  Instructions must state that computer code used to analyse the data must be made openly or publicly available.
5. *Do instructions incentivise making research materials openly available to the research community?*
  Instructions must state that materials used to conduct the research must be made openly or publicly available. Examples include but are not limited to supplemental appendices, questionnaires, survey instruments, scoring rubrics, visual stimuli, and scripts used by research personnel.
6. *Do instructions incentivise the discouragement of publication metrics?*
  Instructions must state that publication metrics (e.g., impact factor, H-index) should not be used.
7. *Do instructions incentivise research quality (e.g., adherence to reporting guidelines)?*
  Instructions must state at least 1 mechanism to promote research quality that was not part of former criteria. Examples include but are not limited to adhering to reporting guidelines, adhering to ethical standards, avoiding or acknowledging biases, prioritising robust methodology, broadening research dissemination, and maximising the value and impact of all research output.
8. *Do instructions incentivise collaboration with a statistician?*
  Instructions must state that applicants consult a statistician for complex quantitative analyses.
9. *Do instructions incentivise any other responsible research practices?*
  Instructions must state any at least 1 other responsible research practice that was not covered in Question 7. Examples include but are not limited to research training, and declaring conflicts of interest.

Full details on definitions of the questions to assess the 9 criteria and definitions of the scores are provided in Additional files 1 and 2. The study protocol and analysis plan, and all data are available on the Open Science Framework (https://osf.io/vnxu6/, https://osf.io/2jesc). A checklist for survey research was used to ensure key information from this audit were reported [14].

## Results

Across the 8 schemes from 5 funders, applicants were instructed or encouraged to address a median of 4 (range 0 to 5) of the 9 criteria (Tables 1 and 2). Three criteria received no mention in any scheme (register analysis protocols, make analysis code open, collaborate with a statistician). With respect to funders, the ARC and NHMRC instructed or encouraged applicants to address 4 or more criteria. However, the smaller funders made no mention of almost all criteria. Applicants were only instructed to register study protocols (NHMRC CTCS), discourage use of publication metrics (NHMRC Project Grants, NHMRC CDF, NHMRC CTCS) and conduct quality research (ARC Discovery Projects, ARC DECRA); other criteria were encouraged but were not required.

**Table 1:** Incentives for responsible research practices in each funding scheme

Do funding instructions incentivise applicants to:	ARC Discovery Projects	ARC DECRA	NHMRC Project Grants	NHMRC CDF	NHMRC CTCS	DART	NBCF	Bupa Health Foundation
1. Publicly register study protocols before starting data collection	No mention	No mention	No mention	No mention	Instructed	No mention	No mention	No mention
2. Register analysis protocols before starting data analysis	No mention	No mention	No mention	No mention	No mention	No mention	No mention	No mention
3. Make study data openly available	Encouraged	Encouraged	Encouraged	Encouraged	Encouraged	No mention	No mention	No mention
4. Make analysis code openly available	No mention	No mention	No mention	No mention	No mention	No mention	No mention	No mention
5. Make research materials openly available	Encouraged	Encouraged	Encouraged	Encouraged	Encouraged	No mention	No mention	No mention
6. Discourage use of publication metrics	No mention	No mention	Instructed	Instructed	Instructed	No mention	No mention	No mention
7. Conduct quality research (e.g. adhere to reporting guidelines)	Instructed	Instructed	Encouraged	Encouraged	Encouraged	Encouraged	No mention	No mention
8. Collaborate with a statistician	No mention	No mention	No mention	No mention	No mention	No mention	No mention	No mention
9. Adhere to other responsible research practices	Encouraged	Encouraged	No mention	No mention	No mention	No mention	No mention	No mention

*ARC* Australian Research Council, *CDF* Career Development Fellowship, *CTCS* Clinical Trials and Cohort Studies, *DART* Diabetes Australia Research Trust General Grants and Millennium Awards, *DECRA* Discovery Early Career Researcher Award, *NBCF* National Breast Cancer Foundation Investigator Initiated Research Scheme, *NHMRC* National Health and Medical Research Council

**Table 2:** Numbers of funding schemes (*n* = 8) that incentivise responsible research practices

Do funding instructions incentivise applicants to:	Instructed	Encouraged	No mention
1. Publicly register study protocols before starting data collection	1	0	7
2. Register analysis protocols before starting data analysis	0	0	8
3. Make study data openly available	0	5	3
4. Make analysis code openly available	0	0	8
5. Make research materials openly available	0	5	3
6. Discourage use of publication metrics	3	0	5
7. Conduct quality research (e.g. adhere to reporting guidelines)	2	4	2
8. Collaborate with a statistician	0	0	8
9. Adhere to other responsible research practices	0	2	6

## Discussion

In our analysis of 8 funding schemes, the schemes only satisfied some criteria on incentives to improve responsible research practices. The ARC and NHMRC were the most robust and stipulated compliance to the Australian Code for the Responsible Conduct of Research. Overall, however, the schemes’ incentives for applicants to implement such practices did not seem strong as they were at most encouraged rather than being required. Grant and fellowship applicants were only instructed to comply with 3 out of 9 criteria in 5 out of 8 schemes. The ability to secure funding and the pressure to publish are potentially strong drivers of research conduct. Consequently, the lack of strong incentives for responsible research practices in funded research may undermine efforts to improve research rigour.

We aimed to assess if Australian health and medical funders incentivise responsible research practices by sampling broadly from the 2018 Australian Competitive Grants Register. To obtain a sample of funding schemes that was feasible to assess yet sufficiently representative of Australian funding schemes, we sampled purposively from the Register, ensuring that schemes from the two major national funders (ARC, NHMRC) as well as smaller funders (e.g., Bupa Foundation) were included. This potentially means our findings are generalisable to the wider population of all schemes for funding health and medical research in Australia.

Our 9 criteria were developed using a selection of recommendations from large consensus discussions on assessing scientists, and implemented principles of research integrity and open science [11, 12]. Although some of our criteria assess reproducible research practices (criteria 1 to 5), others assess broader aspects of research conduct (criteria 6 and 8) and research quality and integrity (criteria 7 and 9). So our criteria assess broad aspects of research integrity and rigour, which extend beyond aspects such as fraud, research reproducibility and open science.

Funders incentivised some responsible research practices (e.g. minimise use of publication metrics, make study data openly available, make research materials openly available) but made no mention of others (e.g. register analysis protocols before starting data analysis, make analysis code openly available, collaborate with a statistician). What might explain these differences? Funders might regard some aspects of responsible research practices as standard practice, and do not see the need to state these explicitly in funding scheme instructions. Or, funders might lack awareness in aspects of responsible research practices and their value. Barriers to implementing incentives for responsible research practices in grant and fellowship schemes may be similar to barriers to adopting open science practices in research publishing. In publishing, limited adoption of open science practices could be due to the rapid need for data (e.g. early during the COVID-19 pandemic), the pressure to publish at high volume and rapidly, potential lack of thoroughness in peer review, and strong institutional incentives for research productivity [15]. In contrast, grant and fellowship applications are reviewed by an expert panel but are never published (to preserve intellectual property and privacy). The limited implementation of incentives for responsible research practices by funders could be due to strong perceptions of what makes grant and fellowship applications “excellent”, such as innovation, significance, and impact. Strengthening the incentives for responsible research practices in funding scheme instructions may require further investigation and joint effort between funders and administering institutions.

Simply encouraging or recommending responsible research practices seems unlikely to substantially change researcher behaviour. For example, journal instructions to authors indicate acceptable standards for publication, and should be used to improve reporting practices. However, audits of papers published before and after the introduction of journal guidelines to improve statistical reporting found no improvement in the proportion of papers reporting statistics correctly [15, 16], or defining statistics correctly in figures [17]. Indeed, a large survey of journals’ instructions to authors across the sciences show that only low to moderate percentages of journals implement transparent reporting practices [18]. Not surprisingly, the quality of published research reports is largely inadequate [19–21]. If most researchers do not implement the recommended reporting practices in journal instructions, it does not seem likely they will implement responsible research practices that are encouraged in funding applications but are not required.

Different strategies could be used to encourage greater uptake of responsible research practices in grant and fellowship funding schemes. First, applicants could be scored and ranked against criteria that strongly incentivise responsible research practices. With competition as a strong driving force, this approach could incentivise new ways to implement these practices, wider implementation, or increased emphasis on aspects of research reproducibility. Second, aspects of responsible research practices could be implemented as criteria for eligibility, with applicants assessed at submission. Third, if applicants are successful, a proportion of funds awarded could be withheld until applicants provide evidence to show they are implementing responsible research practices. The latter two approaches would underscore that responsible research practices are standard procedures in the conduct of research, and may help normalise such research behaviours. Given the recent calls for research reproducibility and transparency [8, 17, 22], it is important that researchers are rewarded for implementing responsible research practices in funded research. This could lead to sustained improvements in the quality of health and medical research.

Some of our criteria would benefit from more nuanced interpretation. In criterion (4), we assessed if funding instructions incentivise applicants to make analysis code openly available. “Analysis code” is often thought to mean computer code, however computer code may not be relevant in some study designs, such as qualitative research or systematic reviews. We could apply a broad interpretation of “analysis code” to mean any method or strategy to analyse data. This would then include methods such as spreadsheets, binary files, or typed/handwritten procedures describing how data were analysed. In criterion (6), we assessed if funding instructions discourage the use of publication metrics. Journal impact factors are often inappropriately used to assess the calibre of individual researchers [23]. The 3 funding schemes that satisfied this criterion specifically stated that impact factors should not be used. In future, this criterion could be refined to refer specifically to impact factors. In criterion (7), we assessed if funding instructions incentivised the conduct of quality research. We applied a broad interpretation of research quality and assessed if funding instructions stated at least 1 mechanism to promote research quality that was not part of former criteria. Examples of these are adhering to reporting guidelines, adhering to ethical standards, and avoiding or acknowledging biases. Thus, this criterion applies broadly to the conduct of research, not only its reporting. In future, this criterion could be refined to include more specific mechanisms to promote research quality, as prompts to auditors. Lastly, in criterion (8), we assessed if funding instructions incentivised applicants to collaborate with a statistician. We included this criterion because statisticians are often involved in research design [24] and as experts in the peer review process [25], but many published studies in our fields still describe common errors from inappropriate statistical techniques. We wanted to assess if funders are aware of these limitations and seek to rectify them. As it is, our study showed that no funding scheme satisfied this criterion. An appropriate methodologist for a study would depend on the study design, and there is a range of methodological expertise across the sciences (e.g. qualitative research, computational modelling, etc.). In future, this criterion could be refined to apply a broader interpretation of methodological expertise.

Our criteria were developed to assess instructions from funding schemes, but they can be adapted and applied in the conduct of research. As an example, we applied the same 9 criteria where relevant in conducting this audit. The study protocol with its methods and analysis plan were publicly registered on the Open Science Framework (OSF) before data collection began. All data and research materials are openly available from the project repository, or in the Additional files 1 and 2 of this manuscript. There was no computer analysis code for this project; data were manually extracted and analysed as outlined in the Methods. We avoided the use of publication metrics in justifying the rationale of this study or discussing our findings. We used a checklist for survey research to ensure that key information were reported. One of the study investigators is a qualified statistician (AB); if this investigator was not involved in the study, we would have sought independent statistical advice had our study required complex statistical analysis and other investigators lacked methodological expertise. One aspect of other responsible research practices is the broad dissemination of research findings to maximise the benefits from research. To implement this aspect of responsible research practice, we chose to submit this work to an open-access journal with a strong focus on research integrity and transparency. Thus, our criteria can be adapted and applied in research studies.

## Conclusions

In summary, Australian grant and fellowship funding schemes incentivise some aspects of responsible research practices, but these incentives could be strengthened. A next step could involve examining our criteria to determine how they could be adapted to suit different funding contexts, barriers to their practical application, and strategies to overcome or mitigate these barriers [26]. Subsequently, funders could then require grant and fellowship applicants to implement responsible research practices. Administering institutions could be required to implement these practices in order to be eligible for funding. These strategies could provide stronger incentives for responsible research practices in funded research, and enhance research rigour. As stated by the NHMRC CEO Professor Anne Kelso, “poor quality publications, data that can’t be understood or replicated won’t change the world … they won’t add to knowledge, they won’t lead to improvements in the length or quality of our lives, and they won’t solve our problems” [27].

## Supplementary Information

**Additional file 1.** Definitions of questions to assess the 9 criteria.

**Additional file 2.** Definitions of scores.

## Data Availability

All data are available from the project repository at the OSF (https://osf.io/vnxu6/). The study protocol and analysis plan were registered on the OSF (https://osf.io/2jesc).

## Abbreviations

ARC: Australian Research Council
CDF: Career Development Fellowship
CTCS: Clinical Trials and Cohort Studies
DART: Diabetes Australia Research Trust General Grants and Millennium Awards
DECRA: Discovery Early Career Researcher Award
NBCF: National Breast Cancer Foundation Investigator Initiated Research Scheme
NHMRC: National Health and Medical Research Council.
R&D: Research and development
