# Supplementary material for: Strengthening the incentives for responsible research practices in Australian health and medical research funding
Source: Res Integr Peer Rev. 2021 Aug 2;6:11. doi: 10.1186/s41073-021-00113-7 (PMC8328133; doi:10.1186/s41073-021-00113-7)
Supplement: Supplementary file 1 — Additional file 1. Definitions of questions to assess the 9 criteria. [file 41073_2021_113_MOESM1_ESM.docx]

**Appendix 1.** Definitions of questions to assess the 9 criteria.

*1. Do instructions incentivise publicly registering study protocols before starting data collection?*

Instructions must state that study protocols must be publicly registered with a date and time-stamp before starting data collection.

For example, only one funding scheme satisfied this criterion. The instructions stated:

Funded clinical trials must be registered in the Australian New Zealand Clinical Trials Registry (ANZCTR) or equivalent before recruitment of the first participant. (NHMRC Clinical Trials and Cohort Studies; CTCS)

The terms “public” and “open” were interpreted as being synonymous. That is, either word is broadly interpreted to mean that there is a public, date and time-stamped record of the information on the internet. The location of this information could include personal websites, third-party repositories, or government sponsored repositories such as [www.clinicaltrials.gov](http://www.clinicaltrials.gov/).

*2. Do instructions incentivise registering analysis protocols before starting data analysis?*

Instructions must state that analysis protocols must be registered with a date and time-stamp before starting data collection.

Instructions would not have satisfied this criterion if they only required researchers to outline how they planned to analyse their data. For example, the statement below would not have satisfied the criterion:

participants must outline briefly in their application how they plan to manage research data arising from a project. (ARC Discovery Projects)

*3. Do instructions incentivise making study data openly available to the research community?*

Instructions must state that data must be made openly or publicly available.

We acknowledge that in some instances, data cannot be shared or made publicly available due to privacy or similar concerns. For example, data from clinical populations could be made available on reasonable request, after administrative approval is granted. If the instructions stated that data should be made openly available but acknowledged that this is not always appropriate, the instructions would have satisfied this criterion.

*4. Do instructions incentivise making analysis code openly available to the research community?*

Instructions must state that computer code used to analyse the data must be made openly or publicly available.

*5. Do instructions incentivise making research materials openly available to the research community?*

Instructions must state that materials used to conduct the research must be made openly or publicly available.

Examples of research materials include, but are not limited to:

- Supplemental appendices
- Questionnaires
- Survey instruments
- Scoring rubrics
- Visual stimuli
- Scripts used by research personnel

*6. Do instructions incentivise the discouragement of publication metrics?*

Instructions must state that publication metrics (e.g. impact factor, H-index) should not be used.

*7. Do instructions incentivise research quality (e.g., adherence to reporting guidelines)?*

Instructions must state at least 1 mechanism to promote research quality that was not part of former criteria. Examples of mechanisms include, but are not limited to:

- Adhering to reporting guidelines
- Adhering to ethical standards
- Avoiding or acknowledging biases
- Prioritising robust methodology
- Broadening research dissemination
- Maximising the value and impact of all research output

*8. Do instructions incentivise collaboration with a statistician?*

Instructions must state that applicants consult a statistician for complex quantitative analyses.

This criterion was included because many published studies in meta-research fields (in which AB and CMK publish) describe common statistical errors made by investigators who fail to apply appropriate statistical techniques in their studies [1–3]. If instructions from the scheme stated that applicants were to include a statistician as a co-investigator, or if the primary applicant was a statistician, this statement would have satisfied the criterion. Ultimately, we wanted to assess whether funding agencies incentivised applicants to implement robust statistical analyses.

*9. Do instructions incentivise any other responsible research practices?*

Instructions must state at least 1 other responsible research practice that was not covered in Question 7. Examples include, but are not limited to:

- Research training
- Declaring conflicts of interest

**References**

[1] Thomas DM, Clark N, Turner D, Siu C, Halliday TM, Hannon BA, et al. Best (but oft-forgotten) practices: Identifying and accounting for regression to the mean in nutrition and obesity research. Am J Clin Nutr 2020;111:256–65. https://doi.org/10.1093/ajcn/nqz196.

[2] Kroeger CM, Ejima K, Hannon BA, Halliday TM, McComb B, Teran-Garcia M, et al. Persistent confusion in nutrition and obesity research about the validity of classic nonparametric tests in the presence of heteroscedasticity: evidence of the problem and valid alternatives. Am J Clin Nutr 2021;113. <https://doi.org/10.1093/ajcn/nqaa357>.

[3] Barnett AG, van der Pols JC, Dobson AJ. Regression to the mean: What it is and how to deal with it. Int J Epidemiol 2005;34:215–20. https://doi.org/10.1093/ije/dyh299.
