# Supplementary material for: Strengthening the incentives for responsible research practices in Australian health and medical research funding
Source: Res Integr Peer Rev. 2021 Aug 2;6:11. doi: 10.1186/s41073-021-00113-7 (PMC8328133; doi:10.1186/s41073-021-00113-7)
Supplement: Supplementary file 2 — Additional file 2. Definitions of scores. [file 41073_2021_113_MOESM2_ESM.docx]

**Appendix 2.** Definitions of scores.

Instructions from the funding scheme guidelines, referenced documents and rubrics that pertained to the audit questions were assessed. For each scheme, criteria were scored as:

- “Yes, applicants are instructed to do so”,
- “Yes, applications are encouraged to do so” or
- “No mention”

Relevant text in response to the criteria were extracted, and are available in the file *data.xlsx* from the Open Science Framework (OSF) project repository (<https://osf.io/vnxu6/>). Examples of language used to differentiate between the scores are reproduced.

“Yes, applicants are instructed to do so”:

Funded clinical trials must be registered in the Australian New Zealand Clinical Trials Registry (ANZCTR) or equivalent before recruitment of the first participant. (NHMRC Clinical Trials and Cohort Studies)

It is not appropriate to use publication metrics such as Journal Impact Factors or the previous Excellence in Research for Australia (ERA) Ranked Journal List when assessing applications. (NHMRC Clinical Trials and Cohort Studies)

“Yes, applicants are encouraged to do so”:

NHMRC supports the sharing of outputs from NHMRC funded research including publications and data. The aims of NHMRC’s Open Access Policy are to mandate the open access sharing of publications and encourage innovative open access to research data. (NHMRC Clinical Trials and Cohort Studies)

We strongly encourage the depositing of data arising from a project in an appropriate publicly accessible discipline and/or institutional repository. (ARC Discovery Projects)

For initial funding schemes during the first round of data collection, criteria were scored using only the instructions from the grant instruction documents and rubrics. However, we noted that the ARC and NHMRC schemes stipulated compliance to several other related instruction and policy documents such as the Code. Consequently, we performed a second round of data collection to extract data from the grant instruction documents and any referenced document. Relevant text satisfying the criteria were extracted *ad verbatim* from the documents to the file *data.xlsx* and the scores were updated on RedCap to reflect information from all documents.
